# Biological Activities of Ribosome-Inactivating Proteins

**DOI:** 10.3390/toxins15010035

**Published:** 2023-01-01

**Authors:** Lucía Citores, José M. Ferreras

**Affiliations:** Department of Biochemistry and Molecular Biology and Physiology, Faculty of Sciences, University of Valladolid, 47011 Valladolid, Spain

After more than 50 years of research, studies on the structure and biological activities of ribosome-inactivating proteins (RIPs) continue to provide a field of great interest within the scientific community, both for the health risks they pose and their applications in medicine and biotechnology. This Special Issue of *Toxins* offers a sample of the main research topics when studying these proteins. RIPs are ribosomal RNA N-glycosylases (EC 3.2.2.22), mainly isolated from plants, some bacteria, and fungi, that specifically catalyze the hydrolysis of the second N-glycosidic bond of the GAGA tetraloop located in the sarcin-ricin loop (SRL) of the major ribosomal RNA. Because SRL is crucial for anchoring elongation factors in the ribosome, the removal of adenine causes the irreversible inactivation of ribosomes, leading to cell death. In addition, RIPs usually demonstrate other enzymatic activities, including, most relevantly, their adenine polynucleotide glycosylase (APG) activity on all nucleic acid types; that is, some RIPs can remove adenines from both ribosomal and non-ribosomal RNA and DNA [1].

RIPs are structurally classified into two groups [1]: type 1 RIPs, consisting of a single polypeptide chain of approximately 30 kDa with enzymatic activity, and type 2 RIPs, of approximately 60 kDa, are formed by an enzymatically active A chain, similar to type 1 RIPs, which is linked through a disulfide bond to a B chain with lectin properties. With a strong affinity for cell surface sugars, the B chain can facilitate toxin entry into cells, thus conferring high toxicity to many type 2 RIPs in cells and animals.

This is the case of ricin and abrin: the first type 2 RIPs described [2]. In medicine, these proteins are mainly used for constructing immunotoxins directed against tumor cells but can also be used as chemical weapons. In this scenario, their most obvious use is in aerosols, which would cause lethal damage to the lungs. Sapoznikov et al. described the effects of abrin and ricin intoxication on the lungs following intranasal exposure in mice [3]. The results indicated that a lethal dose of abrin induced less pronounced damage to the pulmonary stroma and reduced deterioration of intercellular junction molecules compared to ricin, which could contribute to the higher level of protection achieved against abrin by postexposure antibody-mediated treatment.

Although ricin and abrin are the best-known and most used type 2 RIPs, they are not the most toxic. This distinction belongs to RIPs obtained from different species of the genus *Adenia*, such as volkensin, modeccin, lanceolins, and stenodactylin [4]. These proteins differentiate from ricin and abrin due to their unique aspect of being retrogradely transported along peripheral nerves and the central nervous system, providing interesting applications in neuroscience. Bortolotti et al. reported the purification of a new protein of this type from the caudex of *Adenia kirkii* Engl. [5]. Kirkiin is a RIP characterized by high cytotoxicity toward neuronal cell lines, making it a promising candidate for pharmacological purposes.

Type 2 RIPs obtained from species of the genus *Sambucus* are peculiarly hundreds of thousands of times less toxic than ricin and abrin. In the case of elderberry (*Sambucus nigra* L.), more than 20 RIPs and related lectins have been isolated and characterized from its flowers, seeds, fruits, and bark, making it a unique species for studying proteins of this type [6]. The work of Iglesias et al. has expanded our knowledge on the family of RIPs and RIP-related lectins produced by *S. nigra*; their purification and characterization of eight new proteins found in the leaves include one type 2 RIP and two related lectins specific for galactose, four type 2 RIPs with deficient sugar-binding domains, and one type 1 RIP. Several of these proteins are homologous to others found elsewhere in the plant [7].

Type 2 RIPs from *Sambucus* lack toxicity, mainly attributed to a reduced affinity for galactosides which could affect their cell binding, uptake, and the intracellular fate of RIPs. Iglesias et al. compared the binding, endocytosis mechanisms, and intracellular pathway followed by ebulin l (obtained from *Sambucus ebulus* L. leaves) with ricin [8]. The data showed that ebulin l binds to cells less than ricin and how, after binding, ebulin l was taken up by clathrin-dependent and clathrin-independent endocytosis into the endosomal/lysosomal system but not to the Golgi apparatus; importantly, ebulin l did not require clathrin or dynamin for intoxication.

Type 1 RIPs display lower toxicity, as they lack the lectin part and, therefore, cannot bind to cells, as type 2 RIPs demonstrate. The structure of type 1 RIPs is similar to the A-chain of type 2 RIPs, despite differences in the structure of various type 1 RIPs. While RIPs from cucurbits are comparable to the A-chain of type 2 RIPs, RIPs of the pokeweed, carnation, amaranth, and spurge families present greater differences [1]. Monocots, such as those from maize or rice, present the most contrasting RIPs. RIPs from the pokeweed (Phytolaccaceae), carnation (Caryophyllaceae), amaranth (Amaranthaceae), and spurge (Euphorbiaceae) families have been subject to much interest because of their antiviral properties and usefulness for the construction of immunotoxins [9,10]. Four examples of these type 1 RIPs are described in this Special Issue: the curcins from the euphorbiaceous *Jatropha curcas* L. [11], the sodins from the amaranthaceous *Salsola soda* L. [12], the sapovaccarins from the caryophyllaceous *Vaccaria hispanica* (Mill.) Rauschert (= *Saponaria vaccaria* L.) [13], and the PD-Ls from the phytolaccaceaous *Phytolacca dioica* L. [14].

Qin et al. studied the reason behind the differing toxicity of curcin and curcin C on the U20S osteosarcoma cell line [11] and found that curcin C cytotoxicity is higher because, unlike curcin, it is endocytosed by clathrin-dependent endocytosis mediated by LRP1 (low-density lipoprotein receptor-related protein 1): an abundant receptor in this type of cell.

Landi et al. isolated a new type 1 RIP from the seeds, edible leaves, and roots of *Salsola soda* [12]. Sodins showed APG activity and induced apoptosis in Hela and COLO 320 cell lines. Of note, sodin 5, from *S. soda* seeds, and quinoin, from *Chenopodium quinoa* Willd (another amaranthaceous species) seeds showed potent antifungal activity against *Penicillium digitatum* (Pers.) Sacc. [12], making them good candidates for obtaining transgenic plants resistant to fungi.

Schlaak et al. isolated a new type 1 RIP from *S. vaccaria* seeds homologous to type 1 RIPs from other caryophyllaceous that, similar to dianthin 30 and saporin-S6, are used for the construction of immunotoxins [13]. Compared to other type 1 RIPs, they exhibited greater thermostability, suggesting that they would be optimal candidates for targeted cancer therapy.

The exact biological role undertaken by RIPs remains unknown, though it has been considered to mirror a plant defense mechanism against pathogens and predators [1].

Notably, RIPs demonstrate antiviral activity. Their antiviral properties have been investigated for over four decades. However, the emergence of new viruses, giving rise to infectious diseases, has caused interest in these proteins to increase due to the difficulty of treating viral infections. On the other hand, a growing need to control crop diseases without the use of environmentally harmful phytosanitary products has resulted, in this regard, in proving RIPs to be promising tools for obtaining transgenic plants that are resistant to viruses. Citores et al., in this Special Issue, review the research studies addressing this topic, with special emphasis on the latest findings and mechanisms of action proposed [10].

Bulgari et al., using the *Phaseolus vulgaris*-tobacco necrosis virus (TNV) pathosystem, demonstrated that PD-L1 and PD-L4 possess strong antiviral activity [14]. Their experiments suggest that this activity targets viral and ribosomal RNA, explaining the near-complete abolition of infections when the virus and RIP enter the cells together.

The most promising applications of RIPs in experimental medicine, especially in cancer therapy, relate to their use as immunotoxins [15], in which RIPs are linked to antibodies that mediate their binding to and internalization by malignant cells. The main obstacles to treatment with RIPs include their short plasma half-life, nonselective cytotoxicity, and antigenicity. Lu et al. reviewed the strategies used to improve their pharmacological properties and discussed prospects for future developments in the engineering of RIPs [16].

In conclusion, the studies collected in this Special Issue provide the reader with an overview of the most current and interesting lines of research in the field of RIPs, including their applications in medicine and agriculture. Further research on the biological activities of RIPs will allow a greater understanding of their biological role, their more efficient use in medicines, mainly for the treatment of cancer and viral diseases and in the fight against crop diseases caused by viruses, fungi, and insects.
